# Supplementary material for: Content-rich biological network constructed by mining PubMed abstracts
Source: BMC Bioinformatics. 2004 Oct 8;5:147. doi: 10.1186/1471-2105-5-147 (PMC528731; doi:10.1186/1471-2105-5-147)
Supplement: Additional File 2 — The original results of the above study (non-essential files are deleted to keep the file size under the limit set by BMC bioinformatics). [file 1471-2105-5-147-S2.bz2 › chilibotAdditionalFile2/dip10/476ID10734104E10561/html/CCL8_CCRL1.html]

 


 **CCL8** and **CCRL1** 
  
Found 16 abstracts in PubMed, retrieved 10.  
 

 What does Google say? 
 PDF only 
| .edu only 

---

**Interactive relationship** (e.g. stimulation, inhibition, etc)

**Neutral relationship**- Thus, although MCP 2  [ **CCL8** ]  can bind to and signal through CCR1  [ **CCRL1** ] , CCR2b, and CCR5, among which both CCR2 and CCR5 are expressed at high levels on activated T cells, it appears to preferably utilize CCR5 on these cells.  Ref: 9790730 Cell Immunol, 1998

**Non-interactive relationship** (e.g. studied together, co-existance, homology, etc.)

- We used radioligand binding and 35S GTPgammaS exchange assays in membranes from a cell line transfected to express CCR1  [ **CCRL1** ]  Ba F3 hCCR1 to characterize a panel of chemokines HCC 1, MIP 1alpha, MIP 1beta, MIP 1delta, MPIF 1, MCP 2  [ **CCL8** ] , MCP 3, and RANTES as CCR1  [ **CCRL1** ]  ligands.  Ref: 12381680 Br J Pharmacol, 2002
